# Neurological manifestations in HTLV-1 patients with overactive bladder syndrome. A precursor of HAM/TSP?

**DOI:** 10.1186/1742-4690-8-S1-A78

**Published:** 2011-06-06

**Authors:** Davi Costa, André Muniz dos Santos, Néviton Castro, Isadora Siqueira, Edgar Carvalho, Marshall Glesby

**Affiliations:** 1 Immunology, Universidade Federal da Bahia, Salvador, Bahia, Brazil; 2 Infectious Diseases, Cornell Weill Scholl of Medicine, New York, NY, USA

## 

At least 20 million people are infected with HTLV-I virus in the world, and 3 to 5 % develop the classical neurological or hematological manifestations of myelopathy (HAM/TSP) or T-cell leukemia (ATLL). HTLV-I-infected patients with overactive bladder syndrome may represent 37%. We studied 102 HTLV-1 positive individuals without HAM/TSP divided into two groups according to the presence or absence of overactive bladder (OB) syndrome. Individuals with OB were more commonly female (84.3% vs. 60.8% of asymptomatics, P = 0.01), but the mean age in the two groups was similar (46.4 ± 1.9 vs. 42.3 ± 1.7 years, respectively; P = 0.11). The prevalence of neurological complaints was higher in OB group, especially hand or foot numbness and arm or leg weakness. There was no difference between the groups in neurological strength and reflexes. Weakness remained strongly associated with OB in multivariate logistic regression analysis adjusting for sex and age (adjusted odds ratio and 95% CI 3.59(1.45-8.88) in arms and 6.68(2.63-16.93) in legs). In summary, we have characterized a subgroup of HTLV-1 infected patients based on urinary complaints who have more frequent neurological symptoms compared to those without OB. Longitudinal studies are needed to determine if HTLV-1 associated OB is a precursor of HAM/TSP.

